# Impact of binaural beat stimulation on working memory: a graph theory network approach

**DOI:** 10.3389/fnins.2025.1705210

**Published:** 2026-01-08

**Authors:** Muhammad Danish Mujib, Ahmad Zahid Rao, Muhammad Abul Hasan, Ahmad O. Alokaily, Fizza Zia Shaikh, Ahmed A. Aldohbeyb, Saad Ahmed Qazi

**Affiliations:** 1 Department of Biomedical Engineering, NED University of Engineering and Technology, Karachi, Pakistan; 2 Department of Physical Medicine and Rehabilitation, McGovern Medical School, University of Texas Health Science Center at Houston, Houston, TX, United States; 3 Neurocomputation Lab, National Center of Artificial Intelligence, NED University of Engineering and Technology, Karachi, Pakistan; 4 Department of Biomedical Technology, College of Applied Medical Sciences, King Saud University, Riyadh, Saudi Arabia; 5 Department of Electrical Engineering, NED University of Engineering and Technology, Karachi, Pakistan

**Keywords:** binaural-beats, correlation, EEG, graph theory, network properties, working memory

## Abstract

**Introduction:**

Binaural beat (BB) stimulation has been shown to enhance working memory (WM); however, its role in information segregation and neural processing mechanisms remains insufficiently explored. This study hypothesized that improvements in WM recall are associated with enhanced *θ*-band–mediated information segregation in frontal brain regions.

**Methods:**

Sixty healthy participants were randomly assigned to three BB stimulation groups: Group A (*α*-BB, 10 Hz), Group B (*β*-BB, 14 Hz), and Group C (*γ*-BB, 30 Hz). Electroencephalography (EEG; 14 channels, 128 Hz sampling rate) was recorded before (Pre), during (Du), and after (Post) BB stimulation. Cognitive performance was assessed using a digit-span test. EEG power spectra and graph-theoretical network metrics were analyzed across *θ*, *α*, *β*, and *γ* frequency bands. Correlations between EEG measures and cognitive changes were computed, and paired t-tests were used to compare Pre-, Du-, and Post-BB conditions.

**Results:**

*θ*-band activity showed a significant positive correlation with cognitive improvements, particularly in frontal and parietal regions. Group A demonstrated significant increases in *θ*-band clustering coefficient and local efficiency at both global and fronto-parietal network levels, along with enhanced in-degree and out-degree centrality. Group C exhibited increased *θ*-band clustering coefficient during the Post-BB phase and greater betweenness centrality in fronto-parietal regions. No comparable effects were observed in Group B.

**Discussion:**

The findings indicate that BB stimulation, particularly at *α* and *γ* frequencies, enhances WM performance through *θ*-band–mediated improvements in brain network efficiency and information segregation. These results support the potential of BB stimulation as a non-pharmacological approach for cognitive enhancement and provide insight into the neural mechanisms underlying WM modulation.

## 1 Introduction

Working memory (WM) involves a network of brain regions, including prefrontal, parietal, and temporal cortices for encoding and maintaining information (D’esposito et al., 1995; Linden, 2007), and the hippocampus and left inferior frontal gyrus for information retrieval (Öztekin et al., 2009). WM is crucial for reasoning, learning, and comprehension, involving dynamic interactions across brain regions that maintain and manipulate information. Information segregation enables brain areas to specialize in specific information types, while processing integrates these inputs for cognitive tasks. An Electroencephalogram (EEG) is preferred in cognitive neuroscience, providing frequency-specific data on brain connectivity (Luck and Hillyard, 2014; Davidson et al., 2007; Nunez and Srinivasan, 2006). EEG’s noninvasive nature and high temporal resolution make it ideal for studying WM. EEG signals comprise different frequency bands: theta (*θ*, 4–8 Hz), alpha (*α*, 8–12 Hz), beta (*β*, 13–29 Hz), and gamma (*γ*,30–45 Hz).

Studies have reported increased θ- and α-band power in the frontal region, linked to enhanced working memory processes, while γ-band power increases in the occipital region relate to visual WM aspects (Michels et al., 2010; Meltzer et al., 2008). These band power changes may reflect specific brain connectivity patterns associated with WM regions (Linden, 2007; Zanto et al., 2014). Harding et al. (2015) showed the medial prefrontal cortex’s role in directing WM processes and reported increased connectivity from the prefrontal cortex to the parietal lobe. WM training enhanced frontoparietal network connectivity while reducing frontotemporal network connectivity (Jolles et al., 2013; Mujib et al., 2025).

Commonly used noninvasive technologies for WM enhancement include repetitive transcranial magnetic stimulation, transcranial direct current stimulation (tDCS), transcranial alternating current stimulation, neurofeedback, and binaural beats (BB) therapy (Gálvez et al., 2018; Hasan et al., 2023; Mujib et al., 2021; Varastegan et al., 2023; Wang et al., 2024). In BB stimulation, two different auditory tones presented to each ear induce a perceived rhythmic beat at the frequency difference. BB frequencies were selected based on their neurophysiological roles: *α*-BB at 10 Hz for relaxation and cognitive function (D’esposito et al., 1995), *β*-BB at 14 Hz for attention and cognitive performance (Linden, 2007; Beauchene et al., 2017; Beauchene et al., 2016), and *γ*-BB at 30 Hz for cognitive processing and sensory integration (Öztekin et al., 2009). As shown in the EEG network changes, these frequencies modulate fronto-parietal connectivity and facilitate information processing (Beauchene et al., 2017; Beauchene et al., 2016). This phenomenon synchronizes neuronal activity across frequency bands associated with WM-related functions. Studies show BB improves WM performance in healthy individuals and those with cognitive impairments, with changes in temporal, frontal, and parietal regions involved in attention (Kraus and Porubanová, 2015).

Exposure to *α*-BB improves WM (Mujib et al., 2025; Garcia-Argibay et al., 2019) and increases communication efficiency between prefrontal, frontal, parietal, and temporal cortices (Beauchene et al., 2016), which are important for cognitive processing. Studies analyzing brain connectivity following BB stimulation (Mujib et al., 2021; Gao et al., 2014; Solca et al., 2016) have shown higher memory recall related to *θ*-band power and imaginary coherence-based connectivity post-state (Mujib et al., 2021). *α*-BB can modulate interhemispheric connectivity in the temporal lobe (Solca et al., 2016) and increase anterior–posterior intracerebral connectivity in the *θ*-band (Gao et al., 2014). These studies consistently reported increased fronto-parietal connectivity in the θ-band (Mujib et al., 2021; Gao et al., 2014).

Graph theory provides a framework for analyzing brain networks by quantifying information segregation and processing across brain regions. Recent EEG-based graph-theoretical studies reported by Xu et al. (2022) and Wang et al. (2021) have shown that functional connectivity reorganizes dynamically during recovery from mental fatigue and during trust formation. In EEG data, graph metrics such as the cluster coefficient (CC), local efficiency (LE), and centrality reveal how brain regions interact during cognitive tasks. CC measures local connectivity and information segregation (Bassett and Sporns, 2017), while LE indicates network resilience in information transfer (Rubinov and Sporns, 2010). Centrality identifies crucial nodes for global communication and information integration. Moreover, prior research studies (Xu et al., 2022; Xu et al., 2022; Chen et al., 2022; Wang et al., 2020) have shown that graph-theory metrics are highly sensitive to variations in emotional state, performance monitoring, workload, and fatigue. However, the role of graph theory parameters in WM enhancement needs further exploration to understand information processing in the brain (Vecchio et al., 2021; Yaqub et al., 2018).

We hypothesize that WM recall improves with enhanced *θ*-band information segregation in the frontal brain region. We will obtain CC, LE, and centrality measures for θ, *α*, *β*, and *γ* frequency bands during three BB stimulation types. We investigated correlations between the EEG power spectrum and WM task performance.

## 2 Materials and methods

### 2.1 Participants

The study involved 60 healthy individuals (44 males and 16 females), with an average age of 25.73 ± 2.02 years. Participants were divided into three groups: A, B, and C, each comprising 20 participants. *α*-BB at 10 Hz was delivered to Group A (14 males and 6 females), *β*-BB at 14 Hz to Group B (15 males, 5 females), and *γ*-BB at 30 Hz to Group C (15 males and 5 females). All participants followed the same experimental protocol. The study protocols were explained to participants, who provided signed informed consent before experimentation. The Ethical and Advanced Studies Research Committee of NED University of Engineering and Technology approved the research protocol.

The study protocols were explicitly explained to all participants, and informed consent was voluntarily signed and submitted before the session began. The research protocol received approval from the Ethical and Advanced Studies Research Committee of NED University of Engineering and Technology (No. ASRB/877, dated 20-11-2020). The study aimed to contribute to understanding the effects of BB stimulation on brain activity and cognitive performance, potentially providing valuable insights into applications for enhancing working memory.

### 2.2 Experimental procedures

**Figure 1:**
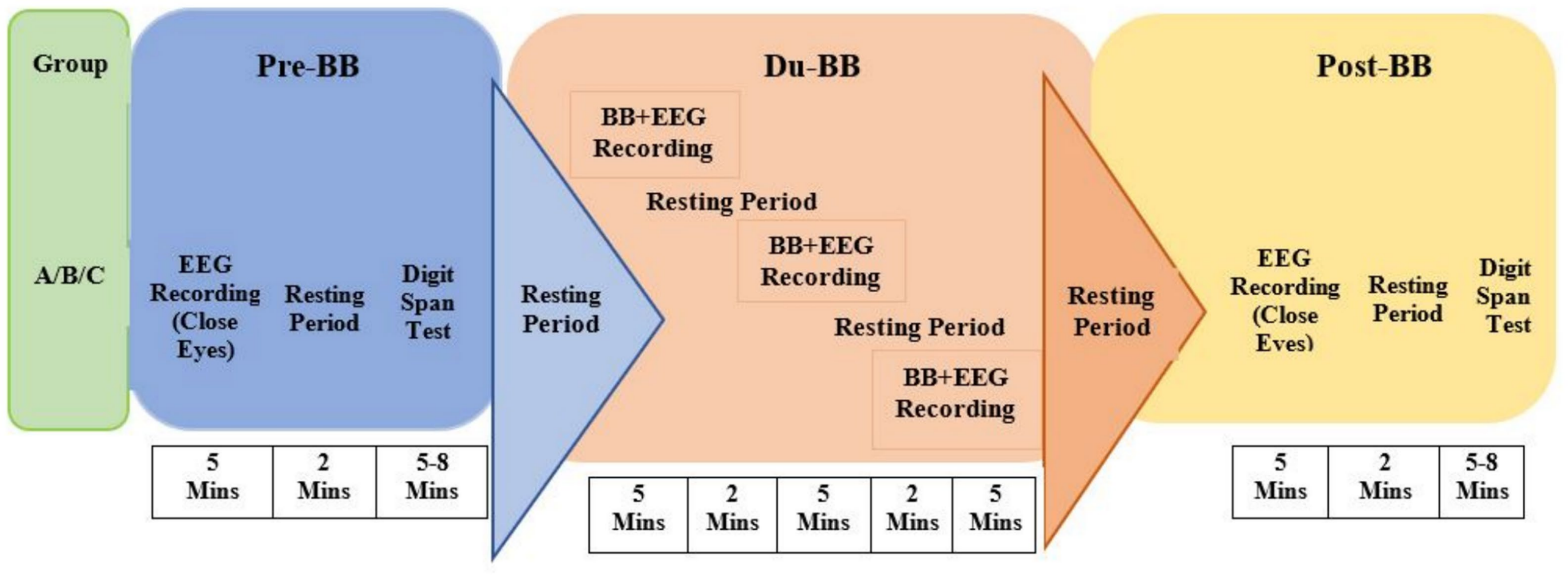
The experimental protocol for BB stimulation was implemented across three groups: group A (*α*-BB), group B (*β*-BB), and group C (*γ*-BB). Pre BB is denoted by pre-BB, during BB by du-BB, and post BB by post BB.

The experimental protocol comprised Pre-BB, Du-BB, and Post-BB (Figure 1). Participants sat comfortably while EEG electrodes were placed on the scalp. The experiment began with a 5-min baseline EEG recording with eyes closed. Sessions were conducted between 10:00 a.m. and 2:00 p.m. to minimize circadian effects on EEG and memory performance. Participants then completed the digit span (pre) test for 5–8 min, repeating number sequences to assess working memory. All three groups underwent BB stimulation in three 5-min sub-sessions, separated by 2-min breaks, with eyes closed. Post-BB EEG was recorded for 5 min in the eyes-closed condition, followed by another digit span test to assess BB frequency-induced brainwave changes.

### 2.3 BB stimulation

The participants were randomly assigned to three BB stimulation conditions: Groups A, B, and C, receiving *α*-BB at 10 Hz, *β*-BB at 14 Hz, and *γ*-BB at 30 Hz, respectively. Pure sinusoidal tones were generated: 10 Hz BB with 410 Hz (right ear) and 400 Hz (left ear); 14 Hz BB with 414 Hz (right ear) and 400 Hz (left ear); and 30 Hz BB with 430 Hz and 400 Hz (right and left ears). The beats were presented for 15 min, divided into three 5-min sub-sessions with 2-min breaks to minimize fatigue. Adobe Audition v3.0 generated these tones as described by Gao and Vernon (Gao et al., 2014; Vernon et al., 2014), while Stereo headphones (MDR-NC7, Sony; Beauchene et al., 2017; Beauchene et al., 2016; Al-Shargie et al., 2019) delivered the stimuli. Participants adjusted volume to a comfortable level, with minimum intensity set at 50 dB above threshold (Al-Shargie et al., 2019). Sessions were standardized for tone duration, volume, and overall duration. In a soundproof room, calibrated headphones measured hearing thresholds using pure tones (400 and 410 Hz for Group A, 400 and 414 Hz for Group B, and 400 and 430 Hz for Group C). Tones started below threshold, increasing by 5 dB until reliable detection. The hearing threshold was recorded as the lowest detectable intensity, with final levels set 50 dB above threshold. Volume adjustments ensured comfortable listening levels.

### 2.4 Digit span test

The participants were shown a random series of digits to complete the digit span test. The test was administered before and after BB stimulation and consisted of five rounds based on the length of a digit sequence. Here, the simplest sequence had four digits in the first round (low level of difficulty) while the most complex consisted of eight digits in the last round. New, random sequences of numbers appeared on the screen each time, for each difficulty level, for both PRBB and POBB states. Participants were instructed to memorize and recall the digits in the sequence of presentation, then enter them using a keyboard immediately after all of the digits were displayed. The maximum number of correctly recalled digits and the time required to complete each round were used to assess participants’ performance. Only those who received a minimum score of 60% were permitted to move on to the following round. The score in each round was obtained by dividing the number of digits sequences properly recalled by the total number of sequences in each trial (Equation 1; Mujib et al., 2021). The final score was determined by dividing the total number of rounds a player completed by the sum of their scores from each round.


(1) $$\;\text{Score}\;(\%)=(\frac{\text{Total number of digits correctly recalled}}{\text{Total number of trials appeared}})\times 100$$

### 2.5 EEG recording

In alignment with the internationally recognized 10–20 system (Jasper, 1958), brain activity from 14 scalp locations (Anterior: AF3, F7, F3, FC5, FC6, F4, F8, AF4 Temporal: T3 and T4, Posterior: P7, O1, O2, P8) was captured using a wireless EEG device called EPOC (Emotiv Technology Inc., United States) in three different states of pre BB (pre-BB), during BB stimulus (Du-BB), and post BB (post-BB). The sampling frequency was set to 128 Hz. For reference, one mastoid (M1) sensor served as the grounding point against which the voltages of all other sensors were measured. Another mastoid (M2) sensor acted as a feedforward reference to minimize external electrical interference, as described by Badcock (Badcock et al., 2013). Electrode impedance was maintained below 5 kΩ by applying saline to the electrodes to enhance conductivity during recording. Data acquisition was performed in a quiet and well-ventilated room.

### 2.6 EEG signal processing

The steps involved in processing EEG data, computing Power Spectral Density (PSD) and absolute coherence, and analyzing brain network properties were as follows:

(1) Artifacts

Signal processing of the EEG data was performed in MATLAB (R2023a, Natick, MA, United States). Baseline correction and DC offset removal were achieved through detrending, while a 5th-order Butterworth band-pass IIR filter (1–45 Hz). The commands “butter (n, Wn, ftype)” and “filter (b, a, x)” were used to calculate filter coefficients and filtered output, where ‘n’ is the filter order, ‘Wn’ is the cutoff frequency, ‘ftype’ specifies the type of the filter, ‘x’ is raw EEG data, ‘b’ and ‘a’ are filter coefficients (Widmann et al., 2015). A visual inspection rejection rate of < 10% was used to ensure accurate identification and elimination of artifacts, including eye blinks, ocular movements, and EMG artifacts, while maintaining signal integrity.

(2) Technique for the power spectrum

The Power Spectral Density (PSD) of the EEG signals was computed using Welch’s method in MATLAB, which smooths the spectrum compared to the raw Fast Fourier Transform (FFT). A 4-s Hanning window with a 2-s overlap was used to minimize leakage and variance. The FFT with 512 points provided a resolution of 0.25 Hz at a sampling rate of 128 Hz. The relative power in the *θ* (4–8 Hz), *α* (8–12 Hz), *β* (13–29 Hz), and *γ* (30–45 Hz) bands was calculated by normalizing the absolute power against the total power from 1 to 45 Hz.

(3) Technique for the power spectrum

Coherence analysis is a widely used technique in neuroscience that quantifies the degree of synchronized activity or oscillatory patterns between different brain regions or electrodes (Bowyer, 2016). The coherence C(f) was calculated using an equation (Equation 2) outlined in Guevara and Corsi-Cabrera (1996).


(2) $$C(f)=(\frac{|CP_{\mathrm{ab}}(f)|2}{\mathrm{Paa}(f)\;x\;\mathrm{Pbb}(f)})$$

Where, C(f)=Coherence betweentwosignals, ${\mathrm{CP}}_{\mathrm{ab}}$ (f) = Cross power spectral density between signals (channels) a and b, $P_{\mathrm{aa}}(f)=\text{Power for signal}\;a,P_{\mathrm{bb}}(f)=\text{Power for signal}\;b.$

For computing $\;CP_{\left(a,b\right)}$ , $P_{\mathrm{aa}}$ and $\;P_{\mathrm{bb}}$ , Welch’s averaged modified periodogram technique was used. A four-second sliding Hanning window with a two-second overlap was used for the analysis. The FFT point count was increased to 512 to achieve a frequency resolution of 0.25 Hz. C(f) has both positive and negative values and can be calculated between all channels of frequencies up to 45 Hz using Equation 1. Thus, C(f) standard procedures were computed in four frequency bands: *θ* (4–8 Hz), *α* (8–12 Hz), *β* (13–29 Hz), and *γ* (30–45 Hz).

### 2.7 Graphical network measurements

Brain regions are exposed to various stimulation frequencies to investigate characteristics. This study analyzed EEG data recorded in different states (Pre-BB, Du-BB, and Post-BB) using graph theory analysis from absolute coherence values. A coherence threshold of 30% was applied to minimize false connections in the brain, ensuring only connections above this threshold were considered. Connectivity measures, including CC, LE, and Centrality, were computed across four frequency bands: *θ* (4–8 Hz), *α* (8–12 Hz), *β* (13–29 Hz), and *γ* (30–45 Hz). The locations of all 14 electrodes were specified based on a 10–20 international electrode placement system. The CC was calculated using the following equation (Equation 3) outlined in Quraan et al. (2013).


(3) $$CC_{i}=\sum (\frac{2t_{i}}{k_{i}(k_{i}-1)})$$

Where, $CC_{i}$ = Cluster coefficient of node i, $k_{i}=\text{denotes}\;a\;\text{degree of}\;\;\text{node}\;i,\text{and}\;t_{i}$ denotes the number of triangles node i.

LE was calculated using equation (Equation 4; Latora and Marchiori, 2001).


(4) $$\mathrm{LE}=\frac{1}{n}\sum LE_{i}=\frac{1}{n}\sum_{i\in N}(\frac{\sum_{j,h\in N,j\neq i}a_{\mathrm{ij}}a_{\mathrm{ih}}{\left[d_{\mathrm{jh}}(N_{i})\right]}^{-1}}{k_{i}(k_{i}-1)})$$

**Figure 2:**
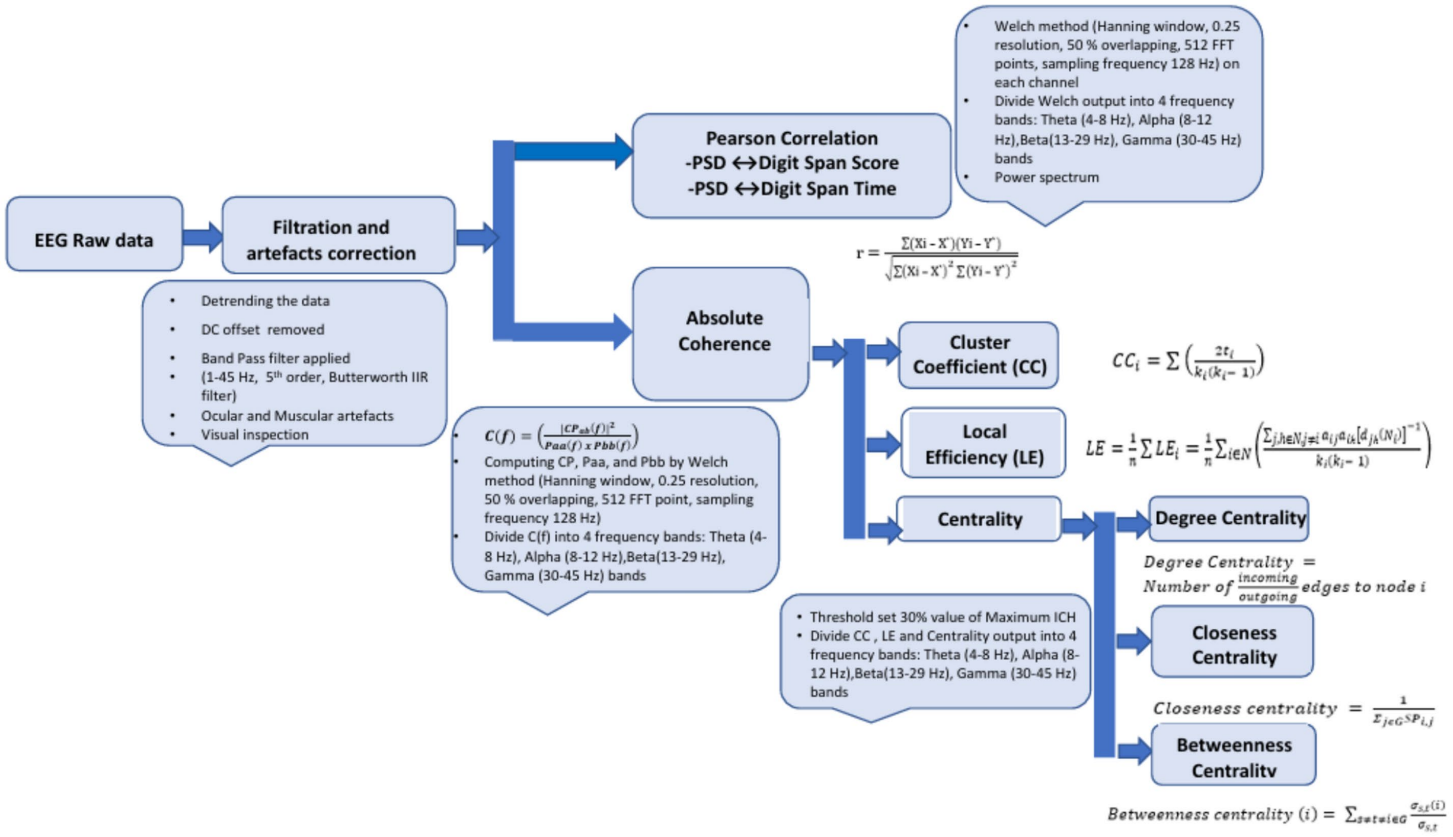
EEG signal processing pipeline: artifact removal, filtering, PSD computation, coherence analysis, and graph theory-based brain network analysis.

Where $LE_{i}$ denotes the local efficiency of node *i*, $a_{\mathrm{ij}}\;$ adjacency matrix entry and $d_{\mathrm{jh}}\;$ denotes the length of the shortest path between *j* and *h* that contains only neighbor of *i* (Figure 2).

### 2.8 Centrality and identification of network hubs

Regional hubs within a network can be identified using various metrics. A high degree is a commonly used criterion. However, various measures of centrality have been advocated for this purpose (Sporns et al., 2007). We used three measures to identify regional hubs: degree, betweenness, and closeness.

(1) Degree centrality

For a directed network, both in-degree (incoming connections) and out-degree (outgoing connections) centrality measures for a node using Equation 5 (Bassett et al., 2008):


(5) $$\text{Degree Centrality}=\text{Number of}\frac{\text{incoming}}{\text{outgoing}}\text{edges to node}\;i$$

(2) Closeness centrality (CN)

Closeness centrality was calculated using equation (Equation 6; Bassett et al., 2008).


(6) $$\text{Closeness centrality}=\frac{1}{\Sigma_{\mathrm{j\epsilon G}}SP_{i,j}}$$

Where j represents one of the nodes that can be reached from the index node i. $SP_{i,j}$ is the shortest path between nodes i and j, and G is the subgraph of nodes and edges connected to node i.

(3) Betweenness centrality.

Betweenness centrality (BW) was calculated using equation (Equation 7; Bassett et al., 2008).


(7) $$\text{Betweenness centrality}\;(i)=\sum_{s\neq t\neq i\in G}\frac{\sigma_{s,t}(i)}{\sigma_{s,t}}$$

Where $\sigma_{s,t}\;$ is the count of shortest geodesic paths between regions s and t, while $\sigma_{s,t}$ (i) is the count of shortest geodesic paths between s and t, which traverse through node i.

### 2.9 Statistical analysis

A two-way ANOVA assessed BB’s impact on cognitive test performance and completion time, with group (A, B, C) and state (pre-BB vs. post-BB) as independent variables, and cognitive scores and task time as dependent variables. After normality testing, Pearson’s correlation was used to examine the relationships between the digit span test and EEG channel power spectral density across four frequency bands for all groups. Statistical significance was set at *p* < 0.05, with analyses performed in MATLAB.

To validate brain state changes, we performed statistical analysis comparing network metrics between Pre-BB, Du-BB, and Post-BB states. We examined CC, LE, In-degree/Out-degree Centrality, Betweenness centrality, and closeness centrality. Paired t-tests assessed significance of differences between states for each metric. Results were adjusted using false discovery rate correction (Benjamini and Yekutieli, 2001) to control Type I error. Cohen’s method calculated effect size to determine practical significance of training effects in Du-BB and Post-BB compared to Pre-state. Effect sizes above 0.8 were large, 0.4–0.8 medium, and below 0.4 small. This revealed a substantial effect size (Cohen, 1988), calculated for both CC and LE changes.

## 3 Results

Demographic analysis showed no age differences among groups (*p* > 0.05), with Group A averaging 25.9 ± 2.02 years, Group B 25.6 ± 2.06 years, and Group C 25.7 ± 2.07 years. Each group had 20 participants, with six females in Group A and five females each in Groups B and C. Pre-BB cognitive scores (Group A: 79.2 ± 7.98, Group B: 79.05 ± 7.14, Group C: 82.22 ± 10.6) and task completion times (Group A: 113.66 ± 12.84 s, Group B: 114.97 ± 8.67 s, Group C: 117.19 ± 11.69 s) were similar (*p* > 0.05). An interim analysis of 10 participants per group, using *p* < 0.025 to avoid false positives, found a large effect size in Group A (d = 0.95 for score, d = 0.926 for time) with high statistical power, while Groups B and C showed smaller effect sizes and lower power. Based on this, 19 participants per group (57 total) were required for the study.

**Figure 3:**
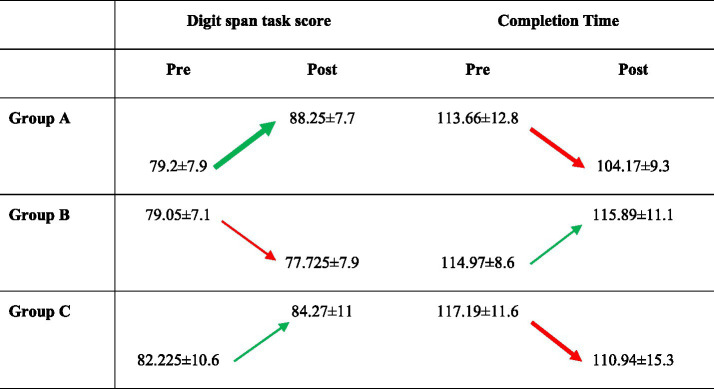
The digit span task scores and completion time among the groups A, B, and C in pre- and post-assessment. Green arrows indicate increases, whereas red arrows indicate decreases in score and time. Significant changes are shown with thick arrows.

Figure 3 presents the comparison of Digit span task scores and Completion time between Pre-BB and Post-BB for the three groups. The Digit span task score (*p* < 0.05, t-value = 5.23) and completion time (*p* = 0.0000157, t-value = − 5.41) showed significant change in the Post-BB with effect size and statistical power (Score: d = 1.15, SP = 0.991 and completion time: *p* < 0.05, *t*-value = − 5.41) in Group A. Group C, which received *γ*-BB, demonstrated a substantial fall in completion time (*p* < 0.05, *t*-value = −2.37), with medium effect size and statistical power (d = 0.46, SP = 0.545). Moreover, Group A showed a significant decrease (*p* < 0.05) in *θ*-band EEG power and increase in *α*-band EEG power. Group C showed a significant increase in θ-band and γ-band power. No significant changes in power were found in Group B.

Table 1 presents the statistical results of the two-way ANOVA. BB stimulation affected scores and task completion time. The main effect of group was significant (*p* < 0.05), and the interaction effect of Group and state (Pre-BB vs. Post-BB) was significant (*p* < 0.05), indicating different effects across groups. The main effect for state was not significant, *F* = 2.74, *p* > 0.05; however, some participants had higher scores post-intervention. For task completion time, effects were significant for groups, *F* = 6.84, *p* < 0.05 and states, *F* = 4.18, *p* < 0.05, showing faster completion post-intervention across groups. The non-significant interaction (*F* = 0.1798) confirmed increased efficiency across groups. BB intervention decreased task time and affected cognitive scores differently by group.

**Table 1:** Two-way ANOVA results for the effects of group, state (pre-BB vs. post-BB), and their interaction on cognitive scores and task completion time.

Source	Score			Time taken		
	F	Prob > F (*p*-value)	η^2^	F	Prob > F (*p*-value)	η^2^
Group	5.12	0.0074	0.0702	6.24	0.0027	0.0844
State	3.31	0.0715	0.0238	5.45	0.0214	0.0387
Group * state	4.54	0.0127	0.0627	1.74	0.1798	0.0251

(1) Correlation between EEG power and digit span test (score and time)

**Figure 4:**
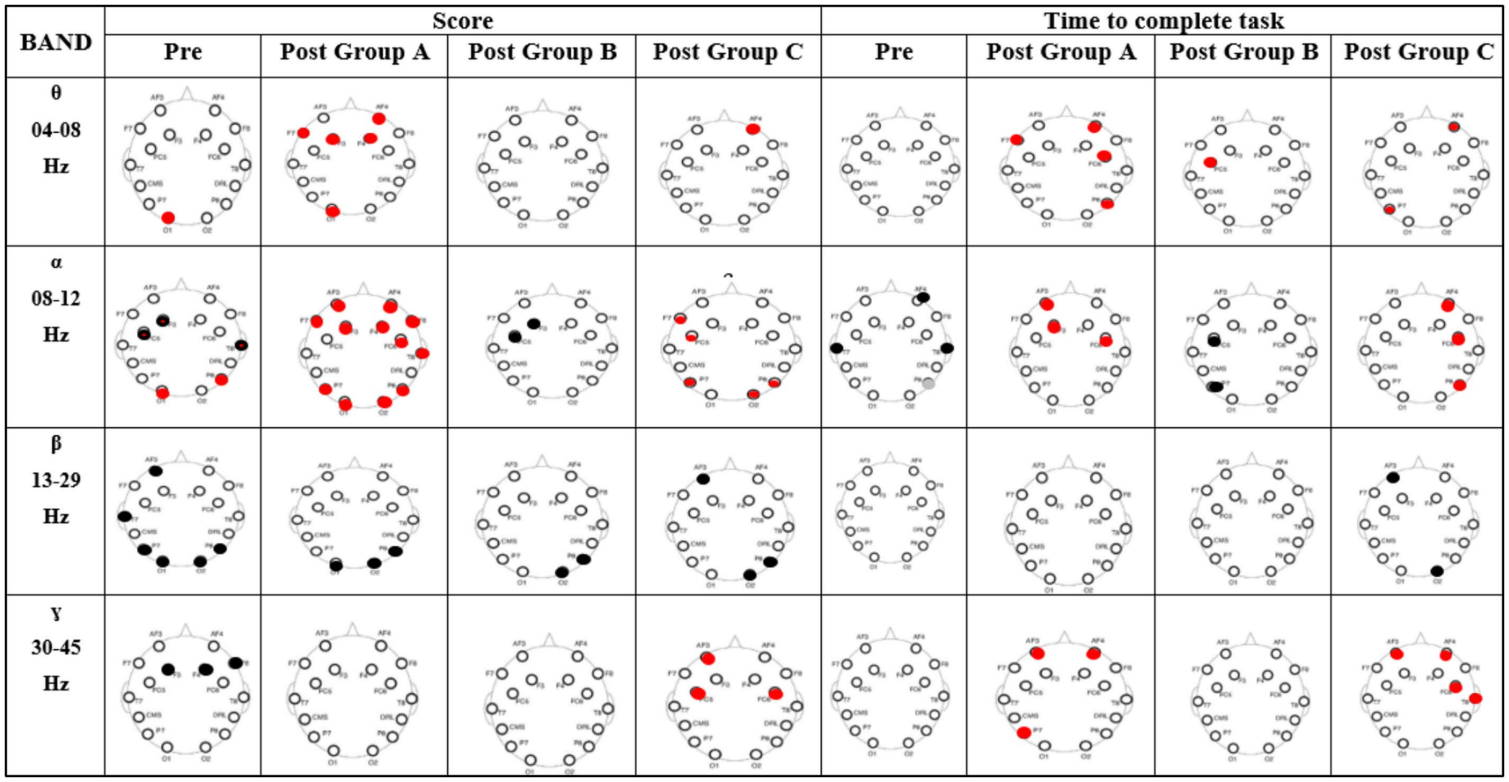
Correlation between digit span task (score and reaction time) and average PSD at each EEG channel for pre- and post-BB states in groups A, B, and C. Columns represent task states and frequency bands (*θ*, α, β, γ); rows show significant correlations—red dots for positive correlation, black dots for negative correlation.

Figure 4 displays the correlation between Digit Span Task performance (score and reaction time) and average PSD of EEG channels for Pre-BB and Post-BB across Groups A, B, and C. The columns represent groups A, B, and C in Pre-BB and Post-BB (scores and time). The rows show significant values between the task and spectral power of EEG channels (gray dots: significant positive correlation, black dots: significant negative correlation). We found positive correlation (*p* < 0.05) in *θ*-band and *α*-band activity in frontal and parietal regions for both score and time in groups A and C. A negative correlation (*p* < 0.05) was found in the *β*-band in the parietal region for scoring. Additionally, the *γ*-band showed positive correlation (*p* < 0.05) in the frontal region for both scoring and timing in Group C, while in Group A, only for scoring. Group B showed a negative correlation (*p* < 0.05) in *α*- and *β*-band activity in frontal and parietal regions for both score and time.

(2) Cluster coefficient and local efficiency

**Figure 5:**
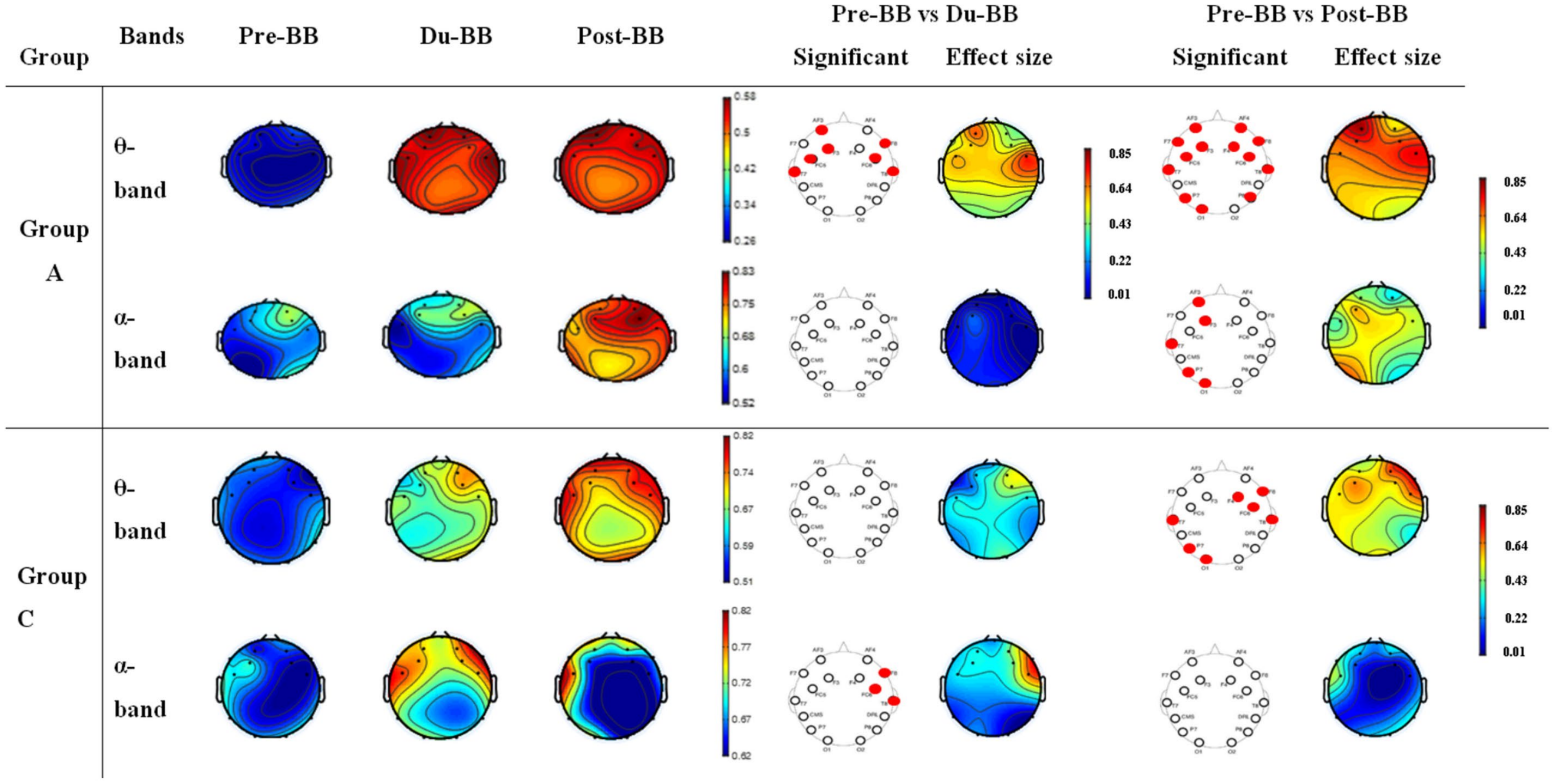
Depicts changes in CC for groups A and C. The first and second rows represent CC changes in the θ- and α-bands, respectively, for group A. The third and fourth rows represent CC changes in θ- and α-bands for group C, respectively. The statistically significant differences between any two states (pre-BB vs. du-BB, and pre-BB vs. post-BB) are shown with filled red (increase value of CC in du-BB and post-BB states as compared to pre-BB) or black (decrease value of CC in du-BB and post-BB states as compared to pre-BB) circles for each particular electrode.

Figure 5 shows CC changes for groups A (first/s rows in *θ*- and *α*-bands) and C (third/fourth rows in *θ*- and α-bands) across Pre-BB, Du-BB, and Post-BB states. Significant differences between states are marked with red circles (increased CC) or black circles (decreased CC) for each electrode. Group A showed significant global CC increases (*p* < 0.05) in *θ*-band during Du-BB and Post-BB states. In the α-band, significant CC and LE increases occurred post-BB in the left hemisphere. No changes were observed in *β*- and *γ*-bands for groups A and C, with no changes in any band for Group B. Group C showed significant global CC increases in the θ-band during Post-BB, while *α*-band increases were limited to the frontal region during Du-BB.

(3) In-degree and out-degree centrality

**Figure 6:**
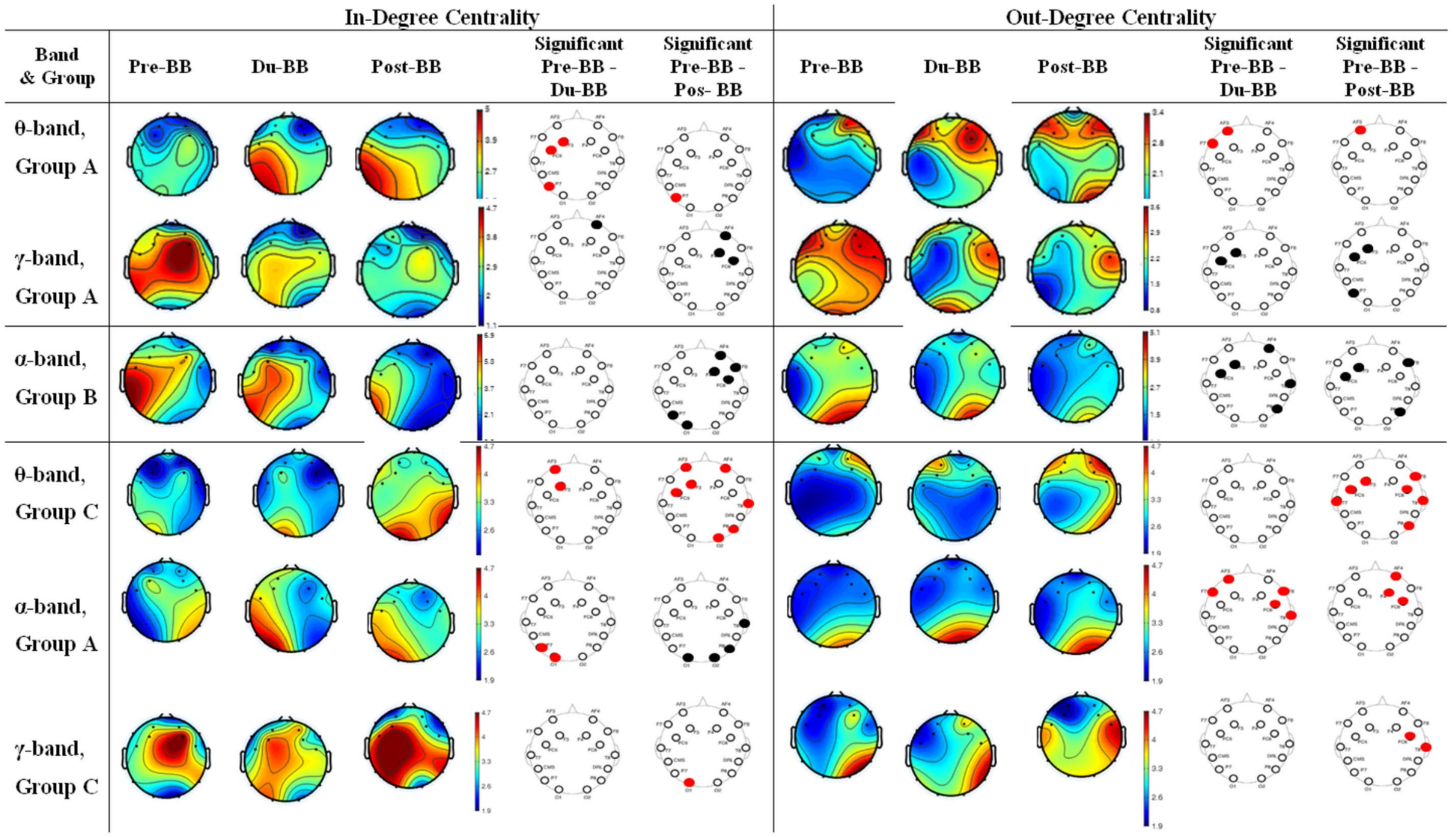
Changes in in-degree and out-degree centrality for groups A, B, and C. The first and second rows represent in-degree and out-degree centrality changes in θ- and γ-bands, respectively, for group A, and the third row represents in-degree and out-degree centrality changes in α-bands. The fourth, fifth, and sixth rows represent changes in in-degree and out-degree centrality in the θ-, α-, and γ-bands, respectively. The statistically significant differences between any two states (pre-BB vs. du-BB, and pre-BB vs. post-BB) are shown with filled red (increase value of in-degree and out-degree centrality in du-BB and post-BB states as compared to pre-BB) or black (decrease value of in-degree and out-degree centrality in du-BB and post-BB states as compared to pre-BB) circles for each particular electrode.

Figure 6 illustrates In-Degree and Out-Degree Centrality for groups A (first and second rows in *θ*- and *γ*-bands), B (third row in *α*-bands), and C (fourth, fifth, and sixth rows in θ-, α-, and *γ*-bands) across Pre-BB, Du-BB, and Post-BB states. Significant differences between states are shown with red (increased centrality) or black (decreased centrality) circles for each electrode. In Group A, *θ*-band showed significantly high in-degree centrality (*p* < 0.05) in fronto-parietal regions, while *γ*-band exhibited significantly low in−/out-degree centrality (*p* < 0.05). No significant changes occurred in *α*- and *β*-bands. Group B showed significantly lower activity between fronto-parietal regions in the α-band, with no changes in other bands. In Group C, fronto-parietal regions showed high in-degree centrality (*p* < 0.05) in the *θ*-band, while parietal regions showed high in-degree centrality in *α*- and *γ*-bands. Regions with high out-degree centrality (*p* < 0.05) were observed in the θ-band, and within frontal and temporal regions in the *α*- and γ-bands. No significant transmission occurred in the β-band.

(4) In-closeness and out-closeness centrality

**Figure 7:**
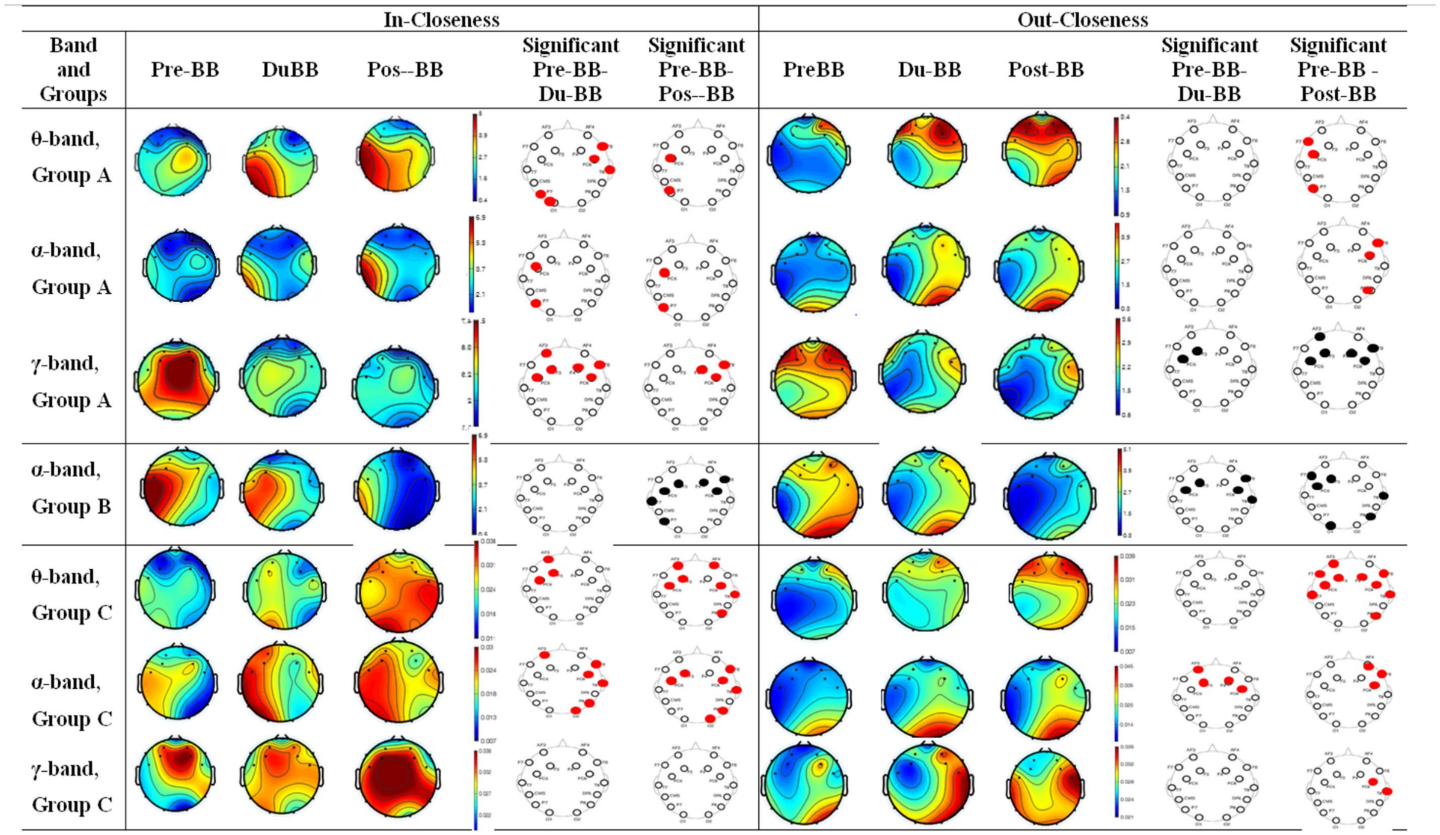
Changes in in-closeness and out-closeness centrality for groups A, B, and C. The first and second rows represent in-closeness and out-closeness centrality changes in the θ- and γ-bands, respectively, for group A; the third row represents in-closeness and out-closeness centrality changes in the α-bands. The fourth, fifth, and sixth rows represent changes in in-closeness and out-closeness centrality across the θ-, α-, and γ-bands, respectively. The statistically significant differences between any two states (pre-BB vs. du-BB, and pre-BB vs. post-BB) are shown with filled red (increase value of in-closeness and out-closeness centrality in du-BB and post-BB states as compared to pre-BB) or black (decrease value of in in-closeness and out-closeness centrality in du-BB and post-BB states as compared to pre-BB) circles for each particular electrode.

Figure 7 shows the-ss and out-closeness centrality for groups A (first, second, third rows in *θ*-, *α*-, *γ*-bands), B (fourth row in α-band), and C (fifth, sixth, seventh rows in θ-, α-, γ-bands) in three states (pre-BB, during-BB, post-BB). Significant differences between states are shown with red (increased values) or black (decreased values) circles for each electrode. Group A showed significantly high (*p* < 0.05) in-and low out-closeness centrality in the frontal region, specifically in the γ-band. In θ- and α-bands, closeness centrality was significantly high (*p* < 0.05) in fronto-parietal region. Group B exhibited lower Closeness Centrality (*p* < 0.05) in the fronto-parietal region in the α-band. Group C displayed high global In-Centrality and Out-Centrality (*p* < 0.05) in frontal regions in *θ*- and *α*-bands. High Closeness Centrality (*p* < 0.05) between frontotemporal regions was observed in *γ*-bands.

(5) Betweenness centrality

**Figure 8:**
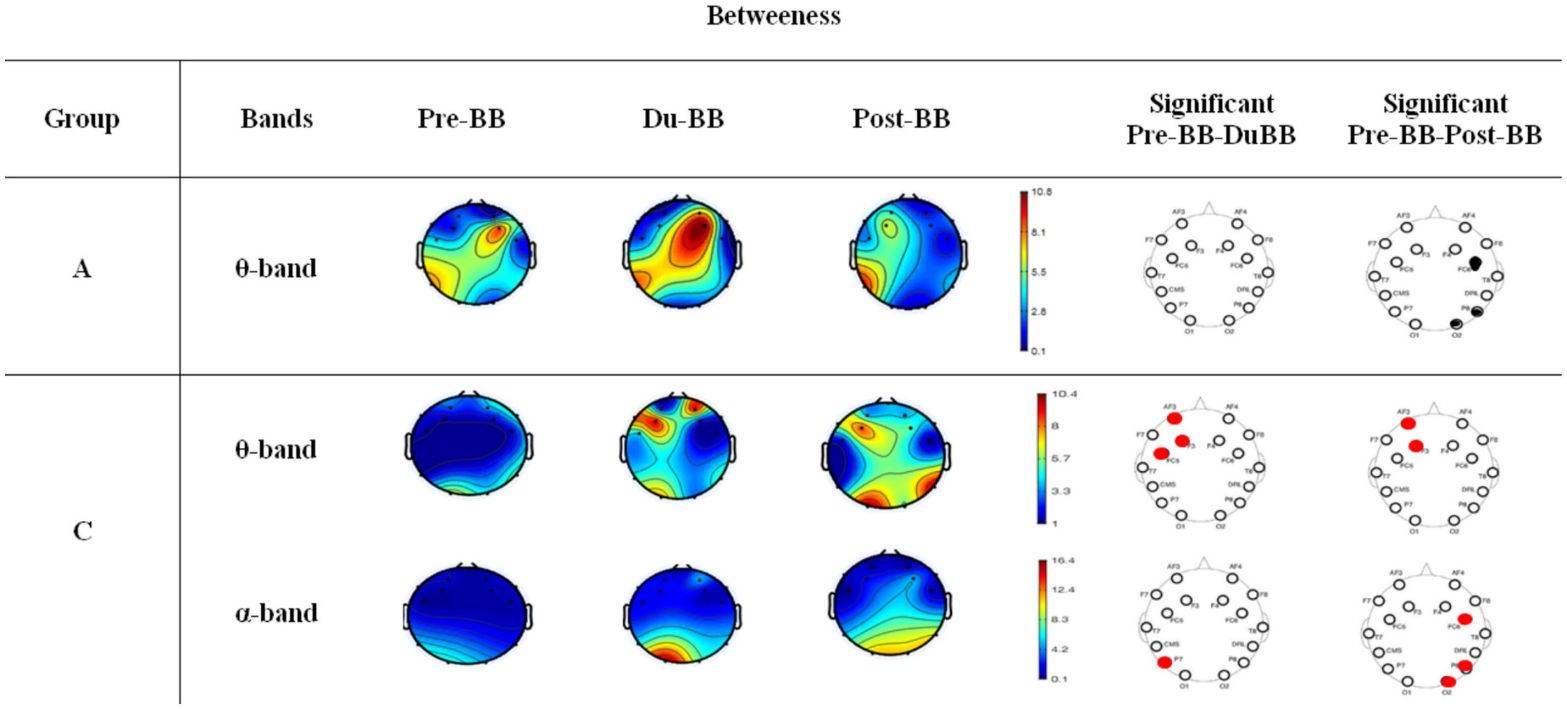
Changes in betweenness centrality for groups A and C. The first row represents changes in betweenness centrality in θ-band for group A, and the second and third rows represent changes in betweenness centrality in θ-and α-bands for group C, respectively. The statistically significant differences between any two states (pre-BB vs. du-BB, and pre-BB vs. post-BB) are shown with filled red (increased value of betweenness centrality in du-BB and post-BB states as compared to pre-BB) or black (decreased value of in betweenness centrality in du-BB and post-BB states as compared to pre-BB) circles for each electrode.

Figure 8 shows Betweenness Centrality for groups A (first row in *θ*-band) and C (second and third rows in θ- and *α*-bands, respectively) in three states (Pre-BB, Du-BB, and Post-BB). The statistically significant differences between any two states (Pre-BB vs. Du-BB, and Pre-BB vs. Post-BB) are shown with filled red (increased value of the Betweenness Centrality in Du-BB and Post-BB states as compared to Pre-BB) or black (decreased value of the Betweenness Centrality in Du-BB and Post-BB states as compared to Pre-BB) circles for each electrode. Group A’s results showed that the frontal and temporal electrodes exhibited significantly high betweenness centrality (*p* < 0.05) in the *θ*-band Du-BB and Post-BB stimulation. However, no significant changes in betweenness centrality were observed in the α-, *β*-, and *γ*-bands, and not in a single band for Group B.

Conversely, Group C revealed that the frontal and parietal electrodes exhibited significantly high (*p* < 0.05) betweenness centrality in the *θ*- and *α*-bands during Du-BB and Post-BB stimulation. However, no significant changes in betweenness centrality were observed in the β- and γ-bands.

## 4 Discussion and conclusion

The present study investigated the effects of BB stimulation on brain network properties and WM, a cognitive process. We hypothesized that improved WM recall tasks would be associated with enhanced information segregation in the θ-band over the frontal region. Results indicate that enhancements in brain network efficiency and cognitive function, especially in the θ-band over the frontal region, suggest BB’s potential as a non-pharmacological intervention for cognitive improvement. Our study revealed correlations between brain activity across frequency bands and regions, demonstrating how BB interacts with the neural mechanisms underlying cognitive processes. Results show a significant positive correlation between *θ*- and *α*-band activities in frontal and parietal regions. This association of θ- and α-activity with cognitive performance, including scoring and task completion time (Mujib et al., 2021; Finnigan and Robertson, 2011; Klimesch, 1999; Alavash et al., 2017; Basharpoor et al., 2021), highlights the role of these oscillations in attentional processes (Alavash et al., 2017; Basharpoor et al., 2021).

The association of frontal and posterior regions for WM processing is well established (Curtis, 2006; Hamidi et al., 2008; Mackey and Curtis, 2017; Eryilmaz et al., 2022; Palva et al., 2010; Takeuchi et al., 2017). BB stimulation may alter neuronal coordination within and between brain regions, potentially improving cognition (Beauchene, 2018; da Silva Junior et al., 2019; Jirakittayakorn and Wongsawat, 2017). Previous studies have primarily focused either on spectral power changes (Gao et al., 2014; Vernon et al., 2014; Jirakittayakorn and Wongsawat, 2017) or on coherence-based connectivity alterations (Mujib et al., 2021; Beauchene et al., 2016; Solca et al., 2016) when investigating the role of binaural beat stimulation. While increases in *θ*- or *α*-band synchrony, along with moderate improvements in memory performance, have been reported in these studies, the joint evaluation of how BB influences both the segregation of local networks and the global information flow has not yet been conducted. In contrast, our study provides a more comprehensive network-level interpretation of BB-induced neural reorganization by integrating PSD changes with graph-theoretical metrics including CC, LE, in−/out-degree, betweenness, and closeness centrality. For instance, Beauchene et al. (Beauchene et al., 2017; Beauchene et al., 2016) demonstrated BB-related increases in connectivity but did not analyze hub dynamics or local efficiency. Similarly, other studies have shown variation in band-specific coherence, dismissing the impact on network topology (Gao et al., 2014; Solca et al., 2016). Compared to these studies, our findings offer deeper mechanistic insight into how *α*-BB and *γ*-BB facilitate working memory processes by revealing frequency-specific enhancement of *θ*-band cluster coefficient and local efficiency, along with reorganization of fronto-parietal hubs. This domain-level comparison not only establishes the novelty of our method but also demonstrates its advantage in quantifying both spectral and topological signatures of cognitive enhancement.

Consistent with this hypothesis, our study revealed increased CC and LE in the θ- and α-bands following BB stimulation (Dai et al., 2017; Langer et al., 2013). Hence, this indicates that BB stimulation, as shown in this study, may enhance information segregation and influence neural activity and communication within the frontal region during WM tasks (Beauchene et al., 2017; Beauchene et al., 2016; Kraus and Porubanová, 2015; Khattak, 2021).

The present findings not only reveal the dominant effects in the theta band but also highlight the significant contributions of alpha and gamma oscillations in shaping the BB-induced enhancement of working memory. Research shows that alpha activity is widely associated with attentional control, inhibition of irrelevant information, and stabilization of task-relevant representations (Zanto et al., 2014; Latora and Marchiori, 2001). The observation in Groups A and C of increased alpha-band activity suggests enhanced top-down regulation during memory processing. On the contrary, gamma-band oscillations are strongly linked to perceptual binding, rapid integration of information, and synchronization of high-frequency within local neural assemblies (Beauchene, 2018; Jensen and Lisman, 1998). Group C showed a significant increase in gamma power and betweenness centrality, indicating enhanced local processing efficiency in fronto-parietal hubs, which likely supports faster and more refined information transmission. Notably, the synergistic interaction by theta and gamma networks’ concurrent reorganization suggests that theta rhythms provide long-range coordination across cortical regions (Finnigan and Robertson, 2011; De Wit et al., 2012), whereas local circuit integration is facilitated by gamma rhythms (Beauchene, 2018; Jensen and Lisman, 1998). As a result of such cross-frequency cooperation, both global segregation and local processing enhance offering a mechanistic explanation for why Group C demonstrated improvement in network efficiency and cognitive performance despite receiving stimulation at higher-frequency.

It is important to consider the biological meaning underlying these graph-theoretical metrics to further clarify their functional relevance. An increase in the cluster coefficient reflects stronger local interconnectedness, indicating more efficient coordination of nearby neuronal populations in segregating task-relevant information, which is a core requirement for the maintenance and encoding of working memory (Xu et al., 2022; Li et al., 2011). Similarly, higher local efficiency suggests enhanced fault-tolerant communication within local circuits, meaning that even with some weak or disrupted connections, information can still be exchanged rapidly (Wang et al., 2021). This is exclusively important in working memory tasks, where robust local processing plays a crucial role in rapid updating and short-term integration of information. Centrality metrics further reveal the extent to which specific brain regions assume hub-like roles; increases in in-degree, out-degree, or betweenness centrality in fronto-parietal nodes indicate that these regions are acting as major relay points for long-range information flow (Badcock et al., 2013; Alavash et al., 2017). These variations imply minimizing communication steps and reducing computational load since the network has reorganized itself to route information more efficiently. The observed increases in CC, LE, and centrality collectively indicate a transition to a more efficient neuronal architecture that facilitates accelerated information transmission, enhanced segregation-integration balance, and superior execution of working memory tasks.

Decision tasks and goal-driven networks can interpret the effects of BB stimulation on WM. Cognitive goals requiring attention, memory retention, and decision-making are achieved through networks that coordinate brain regions (Dagar, 2023). Participants retained numerical sequences through digit span tests, directing goal-directed processes (De Wit et al., 2012). The theta-band involvement hypothesis is supported by increased cluster coefficient and efficiency, showing improved information segregation (Mujib et al., 2025; Klimesch, 1999). BB stimulation may optimize decision-making by strengthening network connectivity, as shown by theta-band activity correlation with improved performance (Mujib et al., 2025; Mujib et al., 2021).

Prior research suggests that neuronal excitability increases during depolarization, leading to synchronous firing and facilitating the coordination and communication between neural networks involved in WM tasks (Jensen and Lisman, 1998; Fell and Axmacher, 2011). This aligns with our results, suggesting that increased CC and LE in the *θ*- and *α*-bands may indicate enhanced depolarization and synchronization of neural activity within functional modules or brain regions. Another study revealed that this neural activity synchronization could lead to more efficient information processing and communication, thereby further contributing to the observed cognitive improvements, specifically in WM (Solca et al., 2016; Perez et al., 2020).

Additionally, the thalamus, a central hub that relays sensory information to different cortices, plays a crucial role in coordinating neural activity (Hwang et al., 2017; Martini et al., 2021). This is because it contributes to the integration of sensory inputs, which are necessary for cognitive processes (Wolff and Vann, 2019). Hence, the observed changes in brain network properties, particularly in the θ- and *γ*-bands, may indicate modulation of thalamo-cortical rhythmicity (thalamic and cortical interactions) and, therefore, could contribute to the improvements in WM performance observed in this and other studies (Moretti et al., 2009; Ribary et al., 2014). Thus, BB has the potential to influence the neural mechanisms. Previous research states that the graph theory network properties utilized for studying information segregation may also be implicated in understanding various cognitive and psychological conditions, such as depression, anxiety, attention problems, and central neuropathic pain (Ewers et al., 2021; Hasan et al., 2021).

Although the findings are promising, further studies are needed to confirm the robustness of the results and assess their sustained long-term effects and implications for clinics. The BB stimulation mechanisms related to thalamocortical rhythmic and depolarization were primarily presented in this study without direct measurements. Therefore, advanced neuroimaging techniques, such as fMRI or high-density EEG, could provide a more comprehensive understanding of these neural processes. Moreover, it may be argued that the study presented findings from only a few electrodes; however, it is important to note that specific brain regions known to be associated with WM processing were chosen for electrode placement.

Group B showed no cognitive improvement despite increases in *θ*- and *γ*-band power. In comparison between the two effective groups, the largest overall effects showing more consistent enhancements in θ- and *α*-band cluster coefficient and local efficiency were demonstrated by Group A whereas, more frequency-specific increases in γ-band centrality and θ–γ coupling were exhibited by Group C.

The neurophysiological role of *β*-oscillations and their limited contribution to working memory mechanisms can be used to explain the absence of cognitive improvement in Group B. β-band activity is not directly related to memory encoding or flexible updating rather it is primarily associated with sensorimotor regulation and maintenance of the current cognitive set (Engel and Fries, 2010; Kilavik et al., 2013). The strengthening of working memory relies on the dynamic interplay between *θ*-based timing mechanisms and α-based inhibitory control, activities that β-frequency entrainment does not consistently stimulate (Kennel et al., 2010; Koochek, 2025). In addition, β-stimulation may induce overly rigid or excessive synchronization, called “β over-binding,” thereby reducing cognitive flexibility. This may prevent the formation of adaptive fronto-parietal hubs required for efficient segregation of information (Spitzer and Haegens, 2017; D’Esposito and Postle, 2015). The Group B confirms that β-BB did not trigger the network-level reorganization, which is necessary for cognitive improvement due to the absence of significant changes in CC, EL, or centrality. Thus, β-BB did not produce functionally meaningful modulation of working memory-related neural circuits in our participants, despite minor increases in *θ*- and *γ*-band power. Even though θ-band activity is strongly associated with WM, we did not include θ-BB stimulation (e.g., 5 Hz) due to the mixed findings in the literature. For instance, various studies have reported no significant cognitive impact from θ-BB stimulation (Garcia-Argibay et al., 2019), suggesting that θ-band activity may be a consequence rather than a driver of cognitive enhancement. Furthermore, θ-BB contradicts the goal of our study because it has been linked to drowsiness and relaxation rather than executive functioning enhancement. By selecting *α*-, *β*-, and γ-BB, we focused on frequencies with well-documented roles in attention, cognitive processing, and working memory enhancement. The BB frequencies associated with Groups A and C had significant effects that facilitated fronto-parietal network adaptations essential for cognitive improvement and thus supported this approach.

While our study provides insights, it has limitations. One concern relates to gender distribution, with 44 males and 16 females in the sample. However, gender bias likely did not influence results since the ratio was maintained across groups, with Group A having 14 males and 6 females, and Groups B and C having 15 males and 5 females. Future studies with larger samples can further confirm the generalizability of our findings.

Another limitation of this study is the absence of a dedicated control group. The inclusion of a control group exposed to white noise strengthened the interpretation of the results by differentiating frequency-specific effects from general auditory stimulation effects. However, our study only compared three different BB frequency conditions. Future research should incorporate such controls to improve the robustness of the findings.

The 14-channel EEG system limits spatial resolution but covers key brain regions involved in WM processing. Future studies may benefit from high-density EEG, functional neuroimaging, or LORETA to refine understanding of BB-induced neural mechanisms. Prior BB studies ensured sufficient exposure while minimizing fatigue, aligning with our 15-min session divided into three 5-min sub-sessions with breaks. Future studies should assess long-term benefits by exploring the effects of prolonged BB exposure.

This study does not directly address clinical conditions but opens potential applications for individuals with mild cognitive impairment, early-stage Alzheimer’s disease, attention deficit disorders, or other cognitive impairments (Mujib et al., 2025; Kennel et al., 2010; Koochek, 2024). These findings provide a foundation for exploring interventions that could be adapted for clinical applications (Hasan et al., 2021; Rao et al., 2024) along with electrical (Mujib et al., 2024), mechanical (Rao and Hasan, 2021), and thermal (Mujib et al., 2023) interventions. Further research should investigate BB stimulation mechanisms in cognitively impaired populations using machine learning (Saif et al., 2021; Rao et al., 2024; Marappan et al., 2022; Rao, 2024), and neurofeedback (Ather, 2024).

Our findings support the hypothesis that enhanced WM recall tasks are linked with increased theta-band information segregation in the frontal region. This is supported by observed changes in theta-band graph theory network metrics, including cluster coefficient and local efficiency. We conclude that BB stimulation may serve as an effective non-pharmacological intervention for cognitive enhancement.

## Data Availability

The raw data supporting the conclusions of this article will be made available by the authors, without undue reservation.
